# Assessing the spatial distribution of methadone clinic clients and their access to treatment

**DOI:** 10.1186/1477-7517-7-14

**Published:** 2010-07-05

**Authors:** Ngai Sze Wong, Shui Shan Lee, Hui Lin

**Affiliations:** 1 Stanley Ho Centre for Emerging Infectious Diseases, The Chinese University of Hong Kong, Prince of Wales Hospital, Shatin, Hong Kong; 2 Institute of Space and Earth Information Science, The Chinese University of Hong Kong, Shatin, Hong Kong

## Abstract

Using Geographic Information System (GIS), the spatial distribution of methadone clinic clients and their utilization of a treatment service in Hong Kong was analysed. A majority (93.7%) of the 63 methadone users recruited were residing in the same district, of which 84.1% spent not more than 15 minutes for traveling. Walking (55.6%) was the commonest transport mode followed by cycling (30.2%). There was no distance decay effect on traveling time, but an association between distance and transport selection could be demonstrated. The residence locations displayed a compact distribution, merging with the general population without any evidence of clustering. Though the distribution of methadone users could have been shaped by the location of clinic, it can also be concluded that methadone clinics at convenient locations are needed if maintenance is a key determinant of service effectiveness.

## Findings

**Figure 1:**
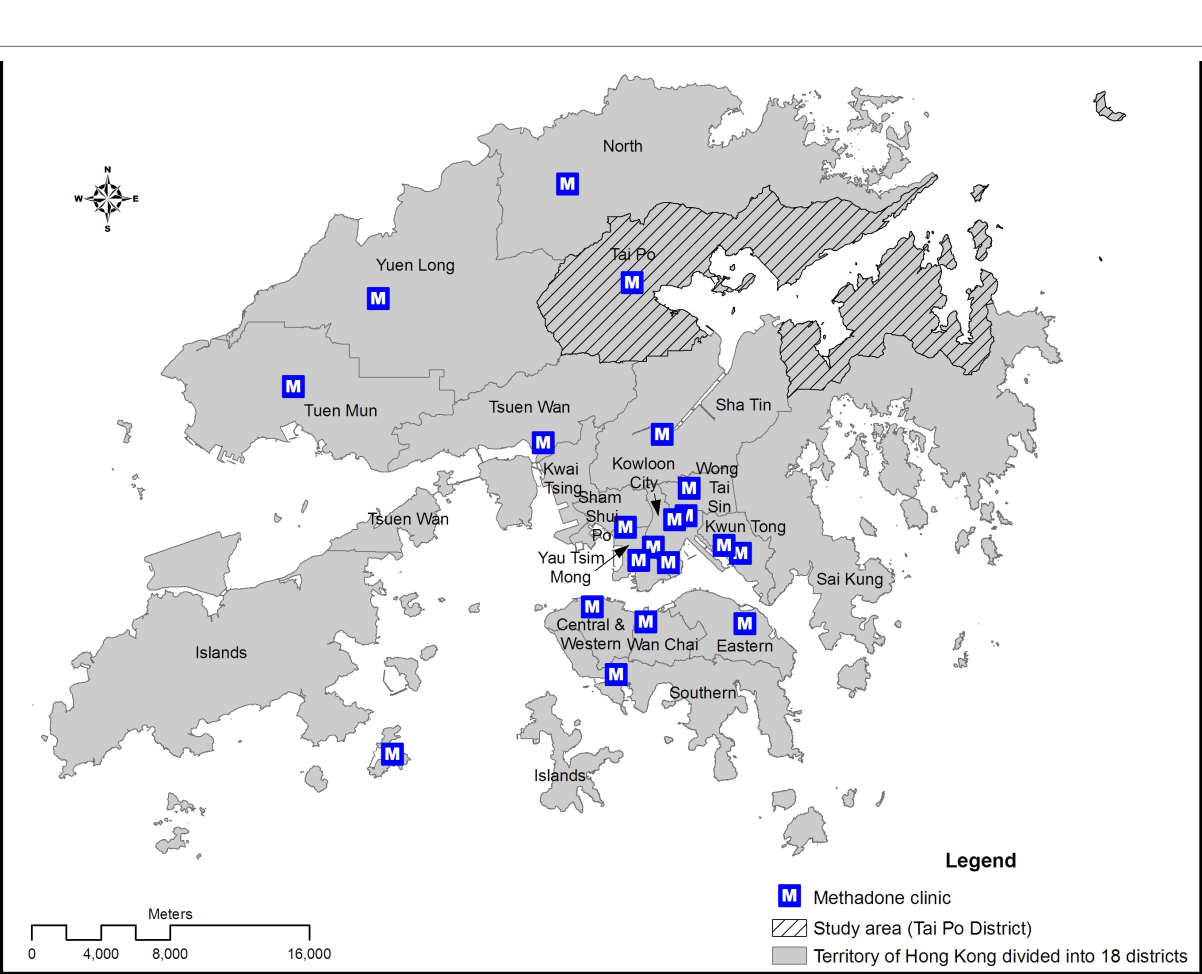
**Map of Hong Kong showing boundaries of 18 administrative districts, location of 20 methadone clinics including the study clinic and the district where the clinic is located**.

Methadone utilization studies have so far been largely conducted on individuals with a cross-sectional approach [1], using national database [2] or through street interviews [3,4]. Apparently, there are knowledge gaps in methadone users' transport selection, association of residence location with distance from clinic, and possible spatial matching between users and the general population. This study aims at determining the spatial pattern of and the spatial influence on methadone treatment utilization in Hong Kong. Located in South Eastern China, Hong Kong has a population of about 7 million, a majority of which Chinese. There are 20 methadone clinics distributing over 16 out of 18 administrative districts,(figure 1) serving more than 95% of heroin users in the territory [5]. Methadone clients were approached by trained interviewers in the vicinity of a clinic located in Tai Po District (figure 1) during its opening hours. Over a one-month period in October 2007, a total of 63 (out of 192 registered) methadone users were interviewed. Questions about utilization included the frequency of visits per week and one's readmission status. Spatial factors included home location, transport mode, total traveling time and transport fare from home to clinic, and residence location. As most methadone users were unwilling to provide specific home address, a relative location estimation method was adopted to supplement the information. Respondents were inquired about location of the nearest convenience store. Buffer was drawn to indicate the most probable location. Estimation was then made by generating random sampling points within the building blocks inside the overlapped buffer areas. Personal factors including gender, age group (<20, 21-40, 41-60 and >60), ethnicity and total number of years on methadone treatment were recorded. GIS software (ArcGIS 9.2) was used for data processing, visualization, and spatial statistical analysis.

Of the methadone users recruited, 39.7% were aged 20-41, 41.3% between 41 and 60, and 19% above 60. The male-to-female ratio (1:0.17) was similar to that of the clinic registrants. In accordance with the clinic's regulation, defaulting treatment for over 28 days requires one to go through a re-registration process, which is defined as "re-admission" in the study. Some 34% of the respondents had not been previously readmitted and had been visiting the clinic every day. This latter sub-group of heroin users was defined as having consistently utilized methadone treatment for maintenance purpose. Geographically, 93.7% of the respondents were living in the district of Tai Po where the clinic is located. Only 3 (4.8%) lived in an adjacent district (North District) and another one further away in Kwun Tong District (figure 1). A couple living in North District went to the clinic every day, explaining that Tai Po clinic was quieter with fewer drug users gathering in its vicinity than the clinic in the district where they resided. As most respondents were local residents, a majority (61.9%) spent 10 minutes or less for going to the clinic. Over 75% spent only 15 minutes for the trip by various transportation mode. The maximum amount of time spent on travelling to the clinic was 60 minutes. Overall, about half (55.6%) went to the clinic by walking and 30.2% by cycling, so they did not need to pay for their daily trip. A minority (14.3%) had to pay for the transportation. Using one-way ANOVA, there was no significant gender difference for the readmission status, F (1, 61) = 2.011, p > 0.05 and the frequency of clinic visits per week, F (1, 61) = 1.898, p > 0.05. Age was not significantly associated with the frequency of clinic visits per week, F (2, 60) = 0.327, p > 0.05. Comparison was made between clients consistently utilizing methadone treatment and otherwise, as defined by daily clinic attendance and absence of readmission. There was no significant difference between the two.

**Figure 2:**
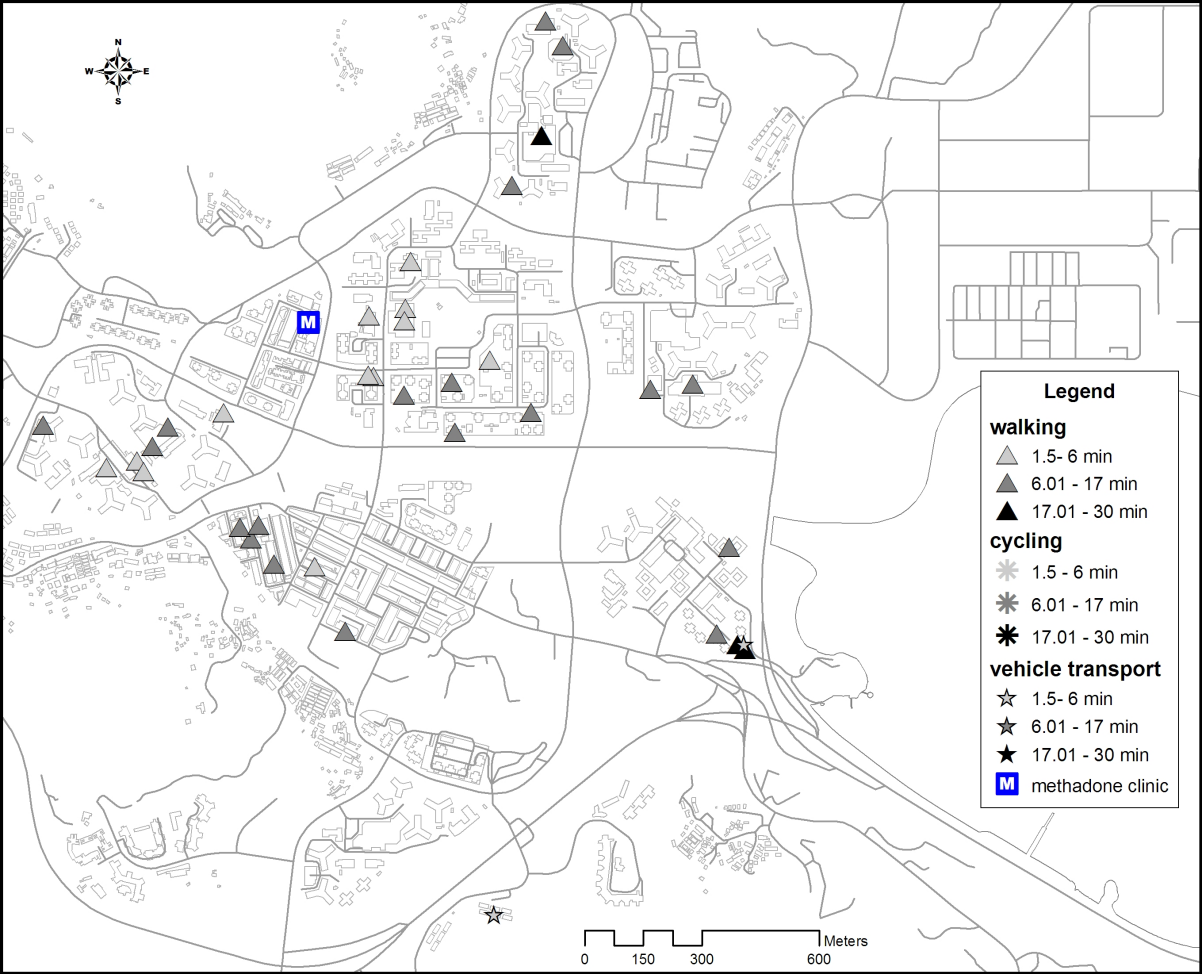
**Transport modes and traveling time of methadone clients, showing resident locations of respondents who attended the clinic on foot, by cycling or vehicle transport, the latter referring to bus, train, taxi and driving (1 patient), and excluding 4 living outside Tai Po District**.

**Figure 3:**
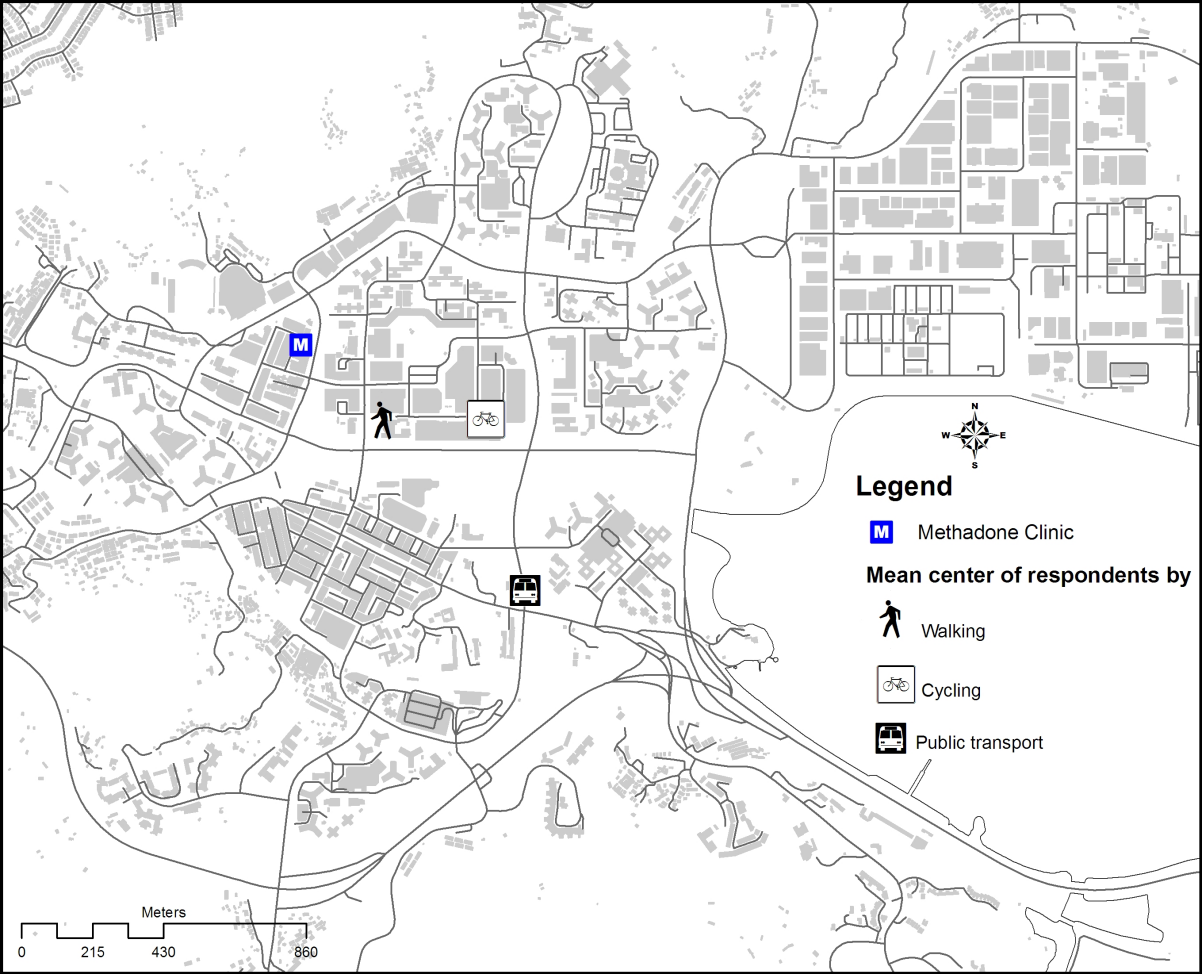
**Mean centres for transport modes of methadone treatment users interviewed in the study**.

**Figure 4:**
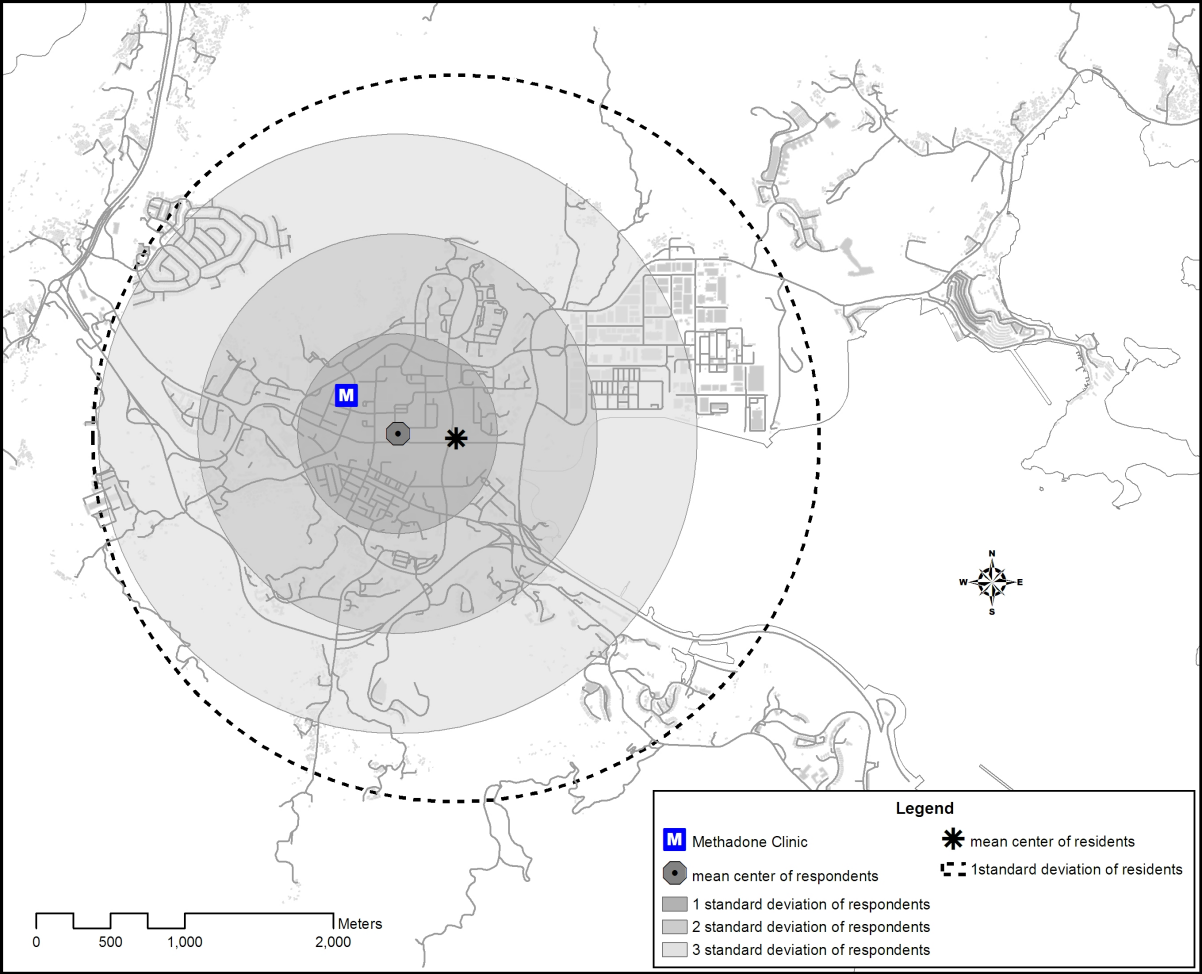
**Comparison of mean centre and standard deviation of residential buildings of Tai Po District and the residence locations of methadone treatment users in the survey**.

Figure 2 shows the spatial distribution of the residence location of respondents living in Tai Po by the relative location estimation method. Figure 3 shows the location of the spatial mean centre [6] for each transportation mode. The mean centre of residence location of respondents choosing the walking mode was closer to the clinic than those coming by cycling. Public transport mode (including bus and mini-bus) gave a mean centre further away from the clinic. Efficiency of walking was reduced by distance friction. Those living farther away were inclined to use more efficient transport mode, such as cycling, to access the clinic. The distribution of Tai Po District's residents was reflected by the mean centre and standard distance of the residential buildings of the district. As illustrated in figure 4, the spatial mean centre of the residence location of respondents living in the district was very close to that of the residential buildings. The methadone clinic was within the buffer zone representing 67% (1 Standard Deviation/S.D.) of the spatial dispersion of local respondents' residence locations. To illustrate the compactness of the distribution, almost all (99.73% or 3 S.D.) local respondents were living in a zone overlapping that of the standard distance (1 S.D.) of residential buildings in the district. In fact none of the local respondents was living beyond the zone covering 1 S.D. of Tai Po residential buildings.

To our knowledge, this is the first spatial study on methadone treatment utilization using GIS applications. The study has enabled us to explore the social and public health contexts of heroin dependence from two inter-relating angles: distribution of methadone clients on one hand, and the utilization of clinic services on the other, as exemplified by the situation in Hong Kong. In interpreting the results in the study, there are two unique features of heroin dependence in Hong Kong which need to be viewed in perspective. First of all, while heroin has continued to be the most important drug of dependence locally, the number of new heroin users has been falling steadily over the years. This is reflected in the statistics of the Central Registry of Drug Abuse, which shows the reported number of new cases falling from 1075 in 2000 to 707 in 2006 [7]. The overall median age of heroin users was high at 41, suggesting that a majority had taken on the habit for years, thus contributing to a high prevalence whereas the incidence has been on the decline. Similar pattern may not be seen in other countries, and the possible impacts on observations made in this study should be interpreted with caution. Secondly, methadone clinics have been in operation in Hong Kong for much longer than their equivalents in neighboring countries. Todate, HIV prevalence has remained low in seroprevalence studies in Hong Kong [8]. Whereas methadone clinics were recently established in response to the rising HIV threats in many Asian countries, the services have been established in Hong Kong before HIV/AIDS was discovered, back in the early 1970s.

Overall, our study suggested a very compact distribution of methadone clients in a district in Hong Kong. The proximity of the mean centre of the respondents' residence locations and that of the district's residential buildings suggested that methadone clients were integrated with the local population. Their location near the District's methadone clinic indicated that they were relatively immobile. This can be explained by the older age of the methadone clients, many of which could have retired and therefore not as active as the younger counterparts. With a median age of 41 in reported heroin users [7], it is possible that the same phenomenon prevailed in other districts in the territory. From another perspective, the distribution of heroin users in Hong Kong might have been influenced by the setting up of methadone clinics about 30 years ago. It can be argued that in order to have good access to methadone, some heroin users might have migrated from their original areas of gathering to the new neighbourhoods of methadone clinics. The compact distribution of heroin users may therefore not be a primary feature of this marginalized community but rather a response to methadone treatment programme. In our study, the traveling time to methadone clinic did not vary significantly with distance from a heroin user's residence location (figure 2). Distance decay was not observed, which could be due to one of 2 reasons: Firstly, all local respondents were living within 1620 m from the clinic. Thus, distance decay might not have started within this range. Secondly, as shown in figure 3, methadone clients living further away tended to use more efficient transport mode to shorten their traveling time, indicating the possible presence of distance decay effect on transport selection rather than travelling time. The influence of distance on transport mode matched with the findings of Field & Briggs on the frequency of visits to GP's surgery in the United Kingdom [9]. Patients living within 1 mile of the facility were more likely to walk while the proportion of those driving to the clinic increased with distance. Our observation carries important implications in the design of methadone treatment services. As utilization hinges on the access and adherence of clients to methadone, having clinics at convenient locations is crucial for ensuring effective maintenance, as has been concluded in other studies [10].

## Competing interests

The authors declare that they have no competing interests.

## Authors' contributions

NSW conducted field study, collated the data, generated the maps, conducted all statistical analyses, and prepared the first draft of the manuscript. HL provided technical advice on the study design. SSL conceptualized the study, coordinated the research and edited the manuscript. All read the revised version of the manuscript and approved the final version.
